# Ferroelectric symmetry-protected multibit memory cell

**DOI:** 10.1038/srep42196

**Published:** 2017-02-08

**Authors:** Laurent Baudry, Igor Lukyanchuk, Valerii M. Vinokur

**Affiliations:** 1 Institute of Electronics, Microelectronics and Nanotechnology (IEMN)-DHS Départment, UMR CNRS 8520, Université des Sciences et Technologies de Lille, 59652 Villeneuve d’Ascq Cedex, France; 2 University of Picardie, Laboratory of Condensed Matter Physics, Amiens, 80039, France; 3 Materials Science Division, Argonne National Laboratory, 9700 S. Cass Avenue, Argonne, Illinois 60637, USA

## Abstract

The tunability of electrical polarization in ferroelectrics is instrumental to their applications in information-storage devices. The existing ferroelectric memory cells are based on the two-level storage capacity with the standard binary logics. However, the latter have reached its fundamental limitations. Here we propose ferroelectric multibit cells (FMBC) utilizing the ability of multiaxial ferroelectric materials to pin the polarization at a sequence of the multistable states. Employing the catastrophe theory principles we show that these states are symmetry-protected against the information loss and thus realize novel topologically-controlled access memory (TAM). Our findings enable developing a platform for the emergent many-valued non-Boolean information technology and target challenges posed by needs of quantum and neuromorphic computing.

The binary information technology reaches its limits set by the atomic size miniaturization and by the fundamental Landauer principle of energy dissipation per bit processing^1^. Employing many-valued logic units, implemented as memory multi-level cells (MLC), reduces energy losses and enables to pack unprecedented high-density information within a single digit overcoming the binary tyranny^2^,^3^,^4^. However, existing implementations of MLC that are currently used in the solid state drives and flash memories, require analogue methods of the bits writing leading to erratic behaviour of the cell due to stochastic loss of information^5^. Here we step in the breach and use an opportunity offered by ferroelectric materials^6^,^7^ that are currently used for implementation of the binary random access memory units^8^. We reach further and utilize the ferrolelectric cubic multiaxiality inherent to ferroelectric perovskite oxides. The target systems are the perovskite thin films, where the substrate-induced strain converts the cubic symmetry to the tetragonal one, enabling the binary up-and-down polarization orientation. However, lifting the cubic degeneracy of the polarization orientations opens yet a new richness of the multi-stable polarization directions (states) in the films, which are not available in the bulk systems. Here we show that these states enable the design of the enhanced performance memory units, ferrolectric multibit cells (FMBC), with the non-trivial topological access to the memory levels by the specific protocol of the applied electric field. The advantage offered by the FMBC as compared to other widely discussed implementations of MLC based on the either of the spin-torque memristive effect^9^, the domain formation in ferroelectric nanodots^10^, the hybrid design^11^,^12^, the DNA-based storage^13^, and the sequential polarization rotation^14^, to name a few, is that while maintaining the simplicity of the material realization, the FMBC ensure the symmetry protection of the memory levels and the strain-temperature programmable architecture of accessing stored information.

## Results

### Multibit hysteresis

**Figure 1:**
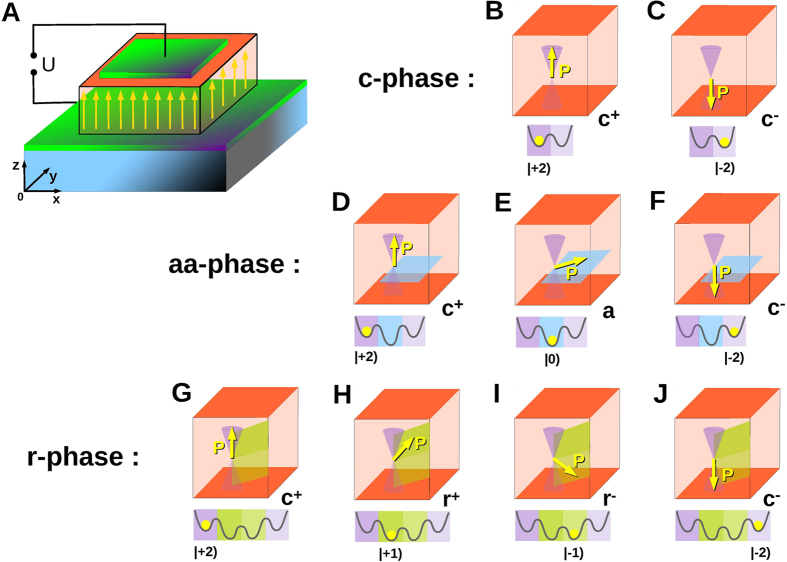
Multistable polarization states in the ferroelectric film. (**A**) Sketch of the experimental setup and coordinate axes (*xyz*). The ferroelectric cell (orange) is grown on the substrate (blue) and is sandwiched between the two electrodes (green). The electric field produced by the voltage operates the polarization orientation. (**B**) and (**C**) The *c*-phase possessing two stable states, *c*^+^ and *c*^−^ of polarization vector, **P**. (**D**) to (**F**) The *aa*-phase having one stable state, *a*, and allowing for two additional metastable states, *c*^+^ and *c*^−^ of **P**. (**G**) to (**J**) The *r*-phase possessing two stable states, *r*^+^ and *r*^−^, and allowing for two additional metastable states, *c*^+^ and *c*^−^ of **P**. The lower sub-panels display the positions of the corresponding polarization states in the minima of the energy relief (yellow spheres) and the respective logical quantum (loq)-numbers.

Shown in Fig. 1 are the experimental setup (A) and the generic ferroelectric phases emerging in strain oxide films (B to J). Each phase is characterized by the stable orientation of the polarization vector, **P** = (*P*_1_, *P*_2_, *P*_3_), where *xyz* components are referred to as {123}, and can possess also several metastable orientations. These phases may be realized in PbTiO_3_^15^ and in Pb(Zr_1−*x*_Ti_*x*_)O_3_ (1 > *x* > 0.4)^16^ at different strains, *u*_*m*_ and temperatures, *T*. The *c*-phase appears, as a rule, at the epitaxial compressive strains and harbors two stable degenerate states, *c*^±^, of *z*-oriented polarization, (0, 0, ±*P*_3_), (Fig. 1B and C). The *aa*-phase, having four stable degenerate states of polarization, (±*P*_1_, ±*P*_1_, 0) oriented along the *xy*-face diagonals of the tetragonal unit cell, appears at high tensile strains. One of these *a*-states with the positive *x* and *y* components of in-plane polarization is shown in Fig. 1E, the others are obtained by the consecutive *C*_4_ rotations over 90° around *z*-axis. In a *r*-phase, appearing at low tensile strains, the equilibrium polarization is directed approximately along one of the space diagonals of the tetrahedral unit cell, hence allowing for eight degenerate orientations (±*P*_1_, ±*P*_1_, ±*P*_3_). Two out of eight *r*-states, denoted as *r*^+^ and *r*^−^, with the positive and negative *z*-components and positive *x*- and *y* components, are displayed in Fig. 1H and I, the others are obtained by *C*_4_ rotations.

We build the memory cells with different number of logic states (energy levels) on the wealth of the above phases. Note, that the *c*-phase, having only two, “up” and “down” oriented polarization states, *c*^−^ and *c*^+^, is a traditional material platform for the binary memory. The promised FMBC with larger number of states adopts *r* and *aa* phases. The key point here is that phases *aa* and *r* maintain not only their inherent stable polarization states, *a* and *r*^±^ respectively, but also can acquire the metastable ones, *c*^±^ (Fig. 1D,F and G,J, respectively). These latter states are the legacy of the polarization stable states of the tetragonal ferroelectric phase that would exist in the bulk material without clamping. Lying in the global and local energy minima, all these stable and metastable states are protected by the pseudo-cubic structure of the system and are thus resistant against the moderate perturbations. As a result, a ferroelectric cell can serve as the symmetry-protected memory storage unit. We quantify the stored information by *logical quantum* (loq)-*numbers*, which are |0) for state *a*, |±1) for states *r*^±^, and |±2) for states *c*^±^ correspondingly. The ordinary binary memory cell constructed from the *c*-phase possesses two loqs |±2) corresponding to *c*^±^ states. The FMBC which we propose are built on the *aa* and *r* phases. The *a*-phase-based FMBC can have three loqs |0) and |±2), corresponding to *a* and *c*^±^ states. The *r*-phase-based FMBC can have four loqs |±1) and |±2), corresponding to *r*^±^ and *c*^±^ states.

**Figure 2:**
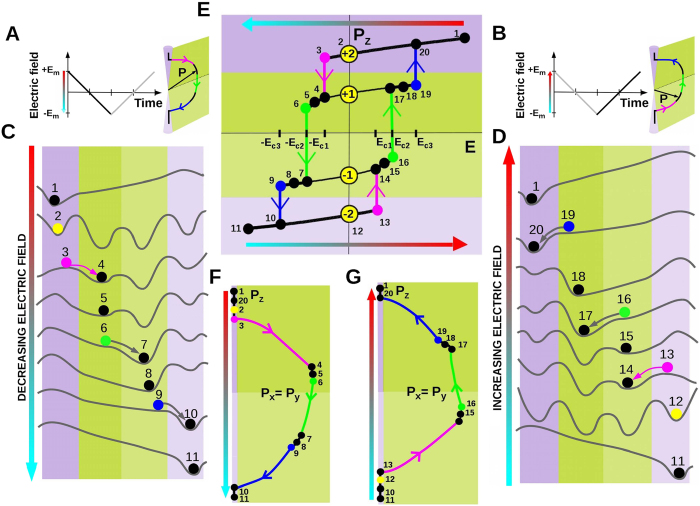
Multibit switching process. (**A**) Positive-to-negative variation of the electric field (left) and the corresponding polarization vector rotation in the *P*_*x*_ = *P*_*y*_ plane (right). **(B)** Reversal negative-to-positive field change and the corresponding polarization rotation. **(C)** Evolution of the potential relief corresponding to varying electric field from +*E*_*m*_ to −*E*_*m*_. Color circles show sequential positions of the system. Polarization passes through all multistable states, *c*^+^, *r*^+^, *r*^−^ and *c*^−^. The point **1** marks the *c*^+^ state, corresponding to the initial positive electric field. Upon decreasing the field below *E* = 0 (**2**) till the critical field −*E*_*c*1_ (**3**) the system looses its stability against the jump to the state *r*^+^ (**4**). It stays in this state (**5**) till another critical field, −*E*_*c*2_ is achieved at point **6** and then jumps down to the state *r*^−^ (**7**). Further field decrease takes the system sequentially through the points **7**, **8**, and **9**. Point **9** corresponds to destabilizing of the state r^−^ at the critical field −*E*_*c*3_. The system falls into the state *c*^−^ (**10**) and stays there till the final negative field (**11**). **(D)** Same as panel **(C)** but under the negative-to-positive field change. Reversing the field variation drives the system back, closing the field cycle at the initial point **1**. **(E)** The multilevel hysteresis loop spanned by the polarization vector upon the positive-to-negative and the reversed variation of the field *E*. It visualizes possible switching between all the four memory logical quantum (loq)-numbers, |±2) and |±1) associated with the states *c*^±^ and *r*^±^ at *E* = 0. The cycle, **1** → **2** →  … → **11** → **12** → …. → **20** → **1**, provides an access to the states with memory logical quantum (loq)-numbers |±2). Access to other loqs, |±1) can be achieved by the partial cycles. For example, the cycle **20** → **2** → **3** → **4** → **17** → **18** → **19** → **20** enables switching between the loqs |+2) and |+1). **(F)** Trajectory of the polarization vector in the plane with *P*_*x*_ = *P*_*y*_ leading to **1** → **11** half-cycle. **(G)** The same as in **(F)** but depicting **11** → **1** half-cycle due to the reversed field variation.

Switching the polarization between the different loqs, hence operating the FMBC, is achieved by applying and then varying the z-aligned electric field, *E*, induced by electrodes. An exemplary operational roadmap for the *r*-phase is shown in Fig. 2. One starts with the complete poling of the FMBC to the up-oriented *c*^+^ state. The gradual decrease of the applied field from the maximal *E*_*m*_ > 0 to minimal −*E*_*m*_ (Fig. 2A) rotates the polarization vector from the up-oriented *c*^+^ state to the down-oriented *c*^−^ state^17^,^18^. The backward field reversely takes **P** to the state *c*^+^ (Fig. 2B).

On its way the polarization repeatedly gets stuck in the energy minima inherited from the equilibrium states *c*^+^, *r*^+^, *r*^−^ and *c*^−^. An example of evolution of the potential relief for **P** under the field-induced bias on the descending branch is shown in Fig. 2C. The transitions between these states occur at critical fields −*E*_*c*1_, −*E*_*c*2_ and −*E*_*c*3_ at which the separating energy barriers vanish. The corresponding evolution for the ascending branch is shown in Fig. 2D. The polarization hysteresis loop, *P*_3_(*E*), realizing the complete *E*_*m*_ → −*E*_*m*_ → *E*_*m*_ field variation cycle and the trajectories of the polarization vector ***P*** are shown in Fig. 2E,F and G. The loop comprises three closed cycles: **20** ↔ **3** → **4** ↔ **19** → **20**; **6** → **7** ↔ **16** → **17** ↔ **6** and **9** → **10** ↔ **13** → **14** ↔ **9**, which take the FMBC to states *c*^+^ (branch **3** ↔ **20**), *r*^+^ (branch **6** ↔ **19**), *r*^−^ (branch **9** ↔ **16**) and *c*^−^ (branch **10** ↔ **13**). Switching off the field when the system moves over one of these branches stalls the memory to the loqs |−2), |−1), |+1), and |+2), respectively. Hereby, constructing an appropriate field variation protocol one gets access to all the four loqs of the multibit memory cell.

### Model

Description of the uniaxially-strained perovskite ferroelectric film, rests on the minimization of the Landau-Devonshire functional (LDF) written in a form proposed in ref. 15


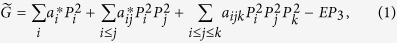


where the 2nd-order coefficients 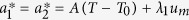 and 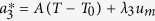 depend on the misfit strain *u*_*m*_ and temperature *T*, and the 4th-order coefficients obey the tetragonal symmetry conditions 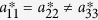, 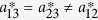. The 6th-order coefficients conserve the cubic symmetry, *a*_111_ = *a*_222_ = *a*_333_, *a*_112_ = *a*_113_ = *a*_223_. The last term in (1) presents the interaction of polarization with electric field. The standard extended form of the LDF (1) and the expression derived from it are presented in the Methods section.

### Energy landscape, bifurcations and catastrophes

The hysteretic jumps between loqs upon the continuous variation of the driving parameter flag the bifurcation-type instabilities which are described and classified by the catastrophe theory^19^,^20^. To describe the switching process we first study the energy landscape of the system under the fixed applied field, *E*. To this end, we minimize the LDF with respect to the position of **P** in the configurational space 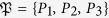. The loci of extrema, {**P**_*λ*_}, *λ* = 1, 2, 3 ... in 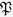 are defined by the condition **J**(**P**_*λ*_, *E*) = 0, where 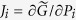 is the Jacobian vector (the so-called Morse points^20^). The extrema in which the Hessian matrix, 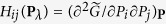 is positively defined correspond to the LDF minima. Varying the field alters the energy relief, in particular, the number and positions of the extrema **P**_*λ*_(*E*) in 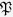. Following the behaviour of a specific minimum, one observes that as soon as the corresponding Hessian matrix ceases to be positive definite at the critical field defined by the condition 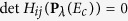 (the non-Morse degenerate point) the bifurcation occurs, the system turns unstable and switches into an adjacent energy favorable state.

Focusing on the switching between the polarization states *r*^±^ and *c*^±^, we reduce the configuration space for the LDF (1) to the 2D plane 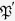  =  {*P*_1_, *P*_1_, *P*_3_} ⊂ 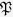, where *P*_1_  =  *P*_2_. To trace hysteresis branches **P**_*λ*_(*E*) ∈ 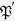 with *λ*  =  {*c*^−^, *r*^−^, *r*^+^, *c*^−^} and determine their critical end-points and corresponding critical fields, we solve the equations for the minima conditions numerically. The advantage of the general catastrophe theory approach is that the type of the catastrophe and the corresponding critical behavior is stable against perturbations, provided the symmetry of the Landau functional preserves^21^. This enables to carry out the complete analysis of the bifurcations at the critical fields *E*_*c*1_, *E*_*c*2_ and *E*_*c*3_ using the LDF (1), linearized in the vicinity of the instability points in 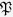 space. The type of the catastrophe is determined by the corresponding catastrophe potential function, *V(p*), where *p* is the deviation of polarization along the instability direction. Thus, the four-fold symmetry of *c*^±^-states imposes the even-terms in the potential, 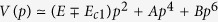 near *E*_*c*1_, resulting in the *butterfly* catastrophe. At the same time, for *E*_*c*2_ and *E*_*c*3_ the generic position of the *r*^±^-states in 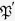 plane yields the *fold* catastrophe with 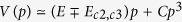. Establishing the type of the catastrophe is important for the correct identification of the thermodynamic criticallity and slowing down of the system, occurring near the instability.

### Switching dynamics in PbTiO_3_

The dynamics of the switching process is described by numerical solution of the time-dependent Landau-Khalatnikov equations 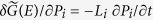, were *L*_*i*_ are the damping coefficients. Let the system be at some arbitrary initial loq. Upon the gradual turn of the electric field, the polarization **P**(*t*) follows quasi-statically the varying *E(t*) and moves along the corresponding hysteresis branch. As the critical non-Morse point is achieved, the instability occurs and the system falls into another state located at the different hysteresis branch. This final state is determined by the time-dependent simulations. Further, turning off the field allows the system to slide along the new hysteresis branch and concludes system’s switching to a new loq.

**Figure 3:**
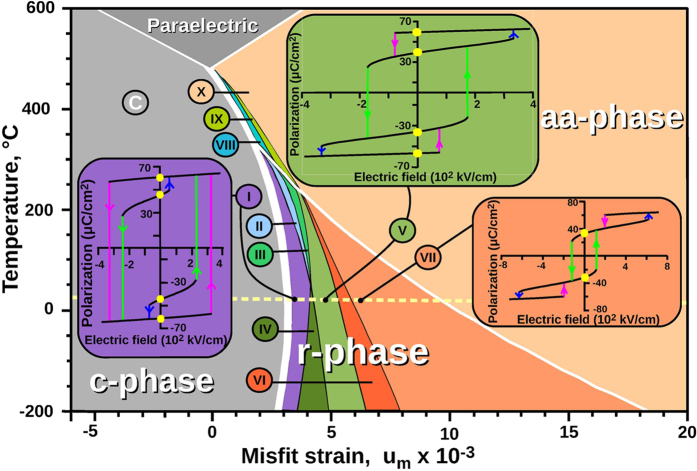
Misfit strain - temperature, *u*_*m*_−*T*, phase diagram of multibit switching regimes in PbTiO^3^ FMBC. The background regions of paraelectric and ferroelectric (*c, r, aa*) phases are those calculated by^15^ and shown in Fig. 1(B) of that paper. Thick white line corresponds to the first order transition between these phases and thin lines stand for the second order transitions. The domains corresponding to different switching regimes are shown as color sectors. The insets display calculated topologically different 4-states hysteresis loops that are realized at room temperature (the room temperature is marked by the dashed yellow line). The loop V corresponds to the 4-loqs (2-bits) fully-switchable FMBC shown in Fig. 2; the loop I displays the 4-loqs FMBC in which 2 loqs are hidden; the loop VII shows the 2-loqs FMBC in which two additional states exist only in non-zero field.

To develop multibit operation principles for FMBC, we investigate into a hysteretic behaviour of the polarization of the strained PbTiO_3_ film in an applied field. To this end we choose the material coefficients in the LDF (1), as proposed in ref. 15, and discuss the behaviour of the system as function of controlling parameters, temperature, *T* and misfit strain, *u*_*m*_. The *u*_*m*_–*T* phase diagram contains three background ferroelectric phases, *c, aa, r*, and paraelectric phase, calculated in ref. 15 (Fig. 3). That the *c*-*r* and *c*-*aa* phase transitions are of the first order, implies that these phases coexist along the transition lines. An applied field lifts the up/down degeneracy of the polarization resulting in the even more rich variety of the coexisting states, *c*^+^, *r*^+^, *r*^−^, and *c*^−^ in the vicinity of the *c*-*r* transition and *c*^+^, *aa*, and *r*^−^ in the vicinity of the *c*-*aa* transition. The interplay between these states gives rise to a remarkable wealth of the switching regimes in *aa*- and *r*-phases, shown as color strips in the Fig. 3. Importantly, the films that realize the FMBC are well screened by the electrodes so that the depolarization field vanishes and the ferroelectric 180^°^ Landau-Kittel domains do not form. At the same time, the films are sufficiently thin, therefore, the substrate-induced strain cannot relax via the ferroelastic domain formation^22^. As a result, the film retains the monodomain state. Moreover, in ultrathin films the critical nucleus does not fit into the film and switching between the monodomain states occurs via the direct polarization turn^23^, bypassing the critical nucleation mechanism^24^.

### Topology of multibit switching

Insets to Fig. 3 show representative examples of the hysteresis loops, corresponding to phases I, V and VII, derived from the described above catastrophe theory analysis of LDF (1) and time-dependent simulations. These topologically different loops are realized in the *r*−phase region at room temperature and at different tensile strains. We start with the description of the 4-loqs loops. The hysteresis loop of type V, which occupies the relatively large strain interval, is already shown in Fig. 2 and discussed above as a typical 4-loqs configuration.

The loop of type I also has 4 loqs, but two of them corresponding to *r*^+^ and *r*^−^ states, |+1) and |−1), are hidden for the repetitive switching. Once the polarization left them, it cannot return back by field variation. It is possible, however, to reach these stable states by the thermal rebooting the system, heating it up to the paraelectric phase and then cooling it back at the zero field. This process represents what we call the hidden-loq memory loops. Finally, the loop VII has only two stable loqs, |+1) and |−1) at *E* = 0 (states *r*^+^ and *r*^−^), whereas two other switchable states, *c*^+^ and *c*^−^, exist only at finite fields.

**Figure 4:**
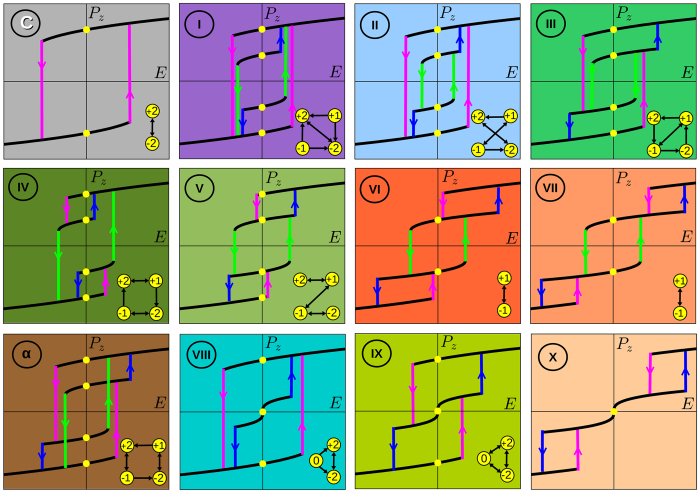
Topologically different multibit hysteresis loops. The number of the panels indicate the region of the phase diagram shown in the Fig. 3. The maps of the switching logical operations are shown in the right bottom corners of the respective panels. C: The traditional one-loop switching between two loqs, |+2) and |−2), the corresponding *c*^+^ and *c*^−^ states are realized in the *c*-phase. I-VII: Multi-loop switching process between states *c*^+^, *r*^+^, *r*^−^, and *c*^−^ in the *r*-phase. Panels I and II correspond to four-loqs unit in which two loqs are hidden. The four-loqs units with topologically-different full access to loqs are shown in panels III, IV and V. Panels VI and VII show the two-loq process in which two more switchable states are accessible in the nonzero field. One more four-loqs process that completes the set of topologically possible configurations is presented in panel *α*. This regime is not realized in the phase diagram of PbTiO_3_ (Fig. 3). The last three panels, VIII, IX, and X enlist the possible switching process in *aa*−phase. Three-loqs loops VIII (with one hidden loq) and IX can serve as platforms for the ternary logical units. The linear sequential configuration IX exhibits properties similar to the recently realized in bismuth ferrite thin films in ref. 14. The double loop X has only one stable loq, but two other switchable states can be realized in nonzero electric fields.

The topologically different hysteresis loops accounting for all combinatorial possibilities of switching procedures are presented in Fig. 4. The richness of the switching protocols rests on possible permutations of the critical fields where the memory states lose their stability (like the already mentioned *E*_c1_, *E*_c2_, and *E*_c3_ in *r* phase). All these processes, except that of panel *α*, are realized in different parts of the *u*_*m*_–*T* phase diagram. The loops are listed in the order of their appearance there, when going from *c*-phase (panel C) to *r*-phase (panels I-VII) and then to *aa*-phase (panels VIII-X). The bottom-right corners of the panels show the corresponding logical operation switching maps. The initial panel C displays the standard generic two-bits hysteresis loop, realized in the *c*-phase with the two available states *c*^+^ and *c*^−^ corresponding to loqs |+2) and |−2). As we have already mentioned, from the topology viewpoint, the switching loops can be divided into three classes. Namely, (i) The full-loqs FMBC (panels III-V, *α* and IX) that allow (re-)switching between all the available states; (ii) The hidden-loqs FMBC (panels I, II and VIII), where two or one states are inaccessible; and (iii) The cells with two (panels VI, VII) and one (panel X) loq(s), respectively, where two additional states arise upon applying an external field.

## Discussion

The proposed FMBC enables the logical operations that are radically different from those provided by the existing MLCs. Namely, the latter allow only for the sequential switching between the available states that can be viewed as a linear one-dimensional chain of events. The ferroelectric multibit cells take all the advantages offered by the 2D topology of the switching maps and, depending on the specific hysteresis loop, can implement different paths of the access to the stored information. For instance, the loop V holds the traditional sequential reversible access from loq |−2) to loq |−1), then, from loq |−1) to loq |+1) etc, whereas in the loop III the loq |+1), is directly accessible from both loq |−1) and loq |−2). We have thus introduced a new type of the topological access memory (TAM), in which the protocol of the access to the symmetry protected quantized logical states can be engineered and tuned by the applied strain and/or temperature.

We have demonstrated that FMBC can be realized using the ultrathin films of ferroelectric oxides. The promising material that provides room temperature operations is a classical ferroelectric PbTiO_3_. One has to ensure that the system falls into the region of the *r*-phase in the phase diagram in Fig. 3, remaining not far from the first-order transition line, where all the variety of the switching regimes occurs. The task can be fulfilled by choosing substrate materials from the family of scandate oxide single crystals, which ensures tailoring of the necessary weak tensile strains^25^. Moreover, it justified theoretically^26^ and demonstrated experimentally^25^ that the 5-nm PbTiO_3_ film is fully coherent with the substrate DyScO_3_ and that no strain-relaxing dislocations and twins form. Thus at room temperature the strained state in the PbTiO_3_ film is in the state compatible with the *r*-phase, in which the polarization deviates away from the tetragonal axis. In this state, the ferroelastic domains are absent and the 180° polarization domains relax off over the time so that the final state appears the monodomain one. The feasibility of realization of four-state sequential loop *V* was also demonstrated recently for multiferroic BiFeO_3_ compound at T = 0 by numerical simulations^27^. A challenging task of inducing the uniform monodomain switching needed for the FMBC, can be addressed through polarization engineering via varying the oxygen chemical potential at the film surface as proposed in the pioneering work^23^ for a similar system PbTiO_3_/SrTiO_3_.

## Methods

The explicit form of the functional (1) is written as:


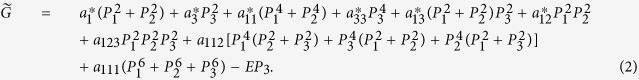


In the plane where *P*_1_ = *P*_2_ we use only two variational parameters, *P*_1_ and *P*_3_, simplifying Eq. (2) to:


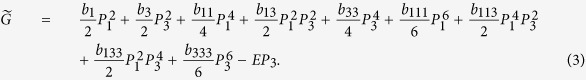


where 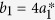, 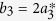, 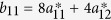, 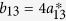, 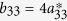, *b*_111_ = 12*a*_111_ + 12*a*_112_, *b*_113_ = 2*a*_123_ + 4*a*_112_, *b*_133_ = 4*a*_112_ and *b*_333_ = 6*a*_111_.

Then, the components of the corresponding Jacobian vector, 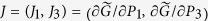, are expressed as:


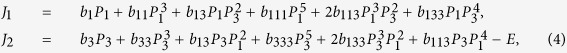


and the corresponding elements of the Hessian matrix 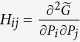 (*i, j* = 1, 3) as:


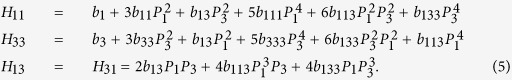


The determinant of the Hessian matrix is calculated as 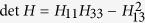.

## Additional Information

**How to cite this article:** Baudry, L. *et al*. Ferroelectric symmetry-protected multibit memory cell. *Sci. Rep.* **7**, 42196; doi: 10.1038/srep42196 (2017).

**Publisher's note:** Springer Nature remains neutral with regard to jurisdictional claims in published maps and institutional affiliations.
